# Anxiety, self-confidence, and self-esteem in relation to competitive coping profiles: a multivariate analysis

**DOI:** 10.3389/fpsyg.2026.1878214

**Published:** 2026-08-12

**Authors:** Chiraz Goumni, Najha Bouallagui, Said Ben Hsan, Raouf Hammami, Riadh Khalifa, Imen Ben Amar

**Affiliations:** 1 Higher Institute of Sport and Physical Education of Ksar-Said, University of Manouba, Manouba, Tunisia; 2 Research Laboratory Sport Performance, Health & Society (LR23JS01), Higher Institute of Sport and Physical Education of Ksar-Said, University of Manouba, Tunis, Tunisia; 3 Tunisian Research Laboratory “Sports Performance Optimization”, National Center of Medicine and Science in Sports (CNMSS), Tunis, Tunisia

**Keywords:** adolescents, behavior, sports psychology, track and field, Tunisian athletes

## Abstract

**Objective:**

This study examined the relationships between self-esteem, competitive state anxiety, and coping strategies in Tunisian junior middle- and long-distance runners, with a focus on gender differences.

**Methods:**

A cross-sectional design was used with 156 athletes (76 males, 80 females; age: 18–19 years). Self-esteem, competitive state anxiety, and coping strategies were assessed using the Rosenberg Self-Esteem Scale, CSAI-2R, and ISCCS. Internal consistency was acceptable to excellent (*α* = 0.70–0.92). Given severe multicollinearity between cognitive and somatic anxiety (*r* = 0.83; VIF = 38.41–39.59), analyses included multivariate analysis of variance (MANOVA) and multiple regression models, complemented by relative importance analysis and bootstrap confidence intervals.

**Results:**

Athletes reported moderate levels of cognitive anxiety (*M* = 12.36 ± 3.57) and somatic anxiety (*M* = 12.97 ± 3.74), and relatively higher self-confidence (*M* = 13.10 ± 3.76). Mental imagery was the most frequently used coping strategy (*M* = 12.00 ± 4.75), whereas social withdrawal was the least used (*M* = 7.38 ± 2.75). Correlation analyses showed that anxiety dimensions were positively associated with maladaptive coping and negatively associated with adaptive coping, whereas self-confidence and self-esteem showed the opposite pattern (all *p* < 0.01). MANOVA indicated a significant multivariate effect of predictors on coping profiles [Wilks’ *Λ* = 0.18, *F*(9, 448.6) = 38.99, *p* < 0.001, *η*^2^_p_ = 0.44], with significant effects for cognitive anxiety (*η*^2^_p_ = 0.10), somatic anxiety (*η*^2^_p_ = 0.06), self-confidence (*η*^2^_p_ = 0.19), and self-esteem (*η*^2^_p_ = 0.29). Regression models explained 71% of the variance in task-oriented coping, 70% in disengagement coping, and 55% in distraction coping. However, multicollinearity between anxiety dimensions resulted in unstable regression coefficients. Relative importance analysis indicated that self-confidence was the strongest predictor of task-oriented (71.3%) and distraction coping (64.6%), whereas self-esteem predicted all coping dimensions (22–25% of explained variance).

**Conclusion:**

Self-confidence and self-esteem are key determinants of coping strategies in junior runners. However, multicollinearity limits interpretation of the unique effects of anxiety dimensions. These findings highlight the importance of psychological interventions aimed at enhancing self-confidence and self-esteem to promote adaptive coping in youth sport.

## Introduction

Despite substantial research examining self-esteem, anxiety, and coping strategies separately in sport psychology, relatively few studies have investigated the interrelationships among these constructs within a single conceptual framework, particularly during adolescence (Nicholls et al., 2009; Tamminen and Gaudreau, 2014). This limitation is important because self-esteem, anxiety, and coping are dynamic and interconnected psychological factors that collectively influence athletes’ adaptation to competitive stress. Understanding how these variables interact may provide a more comprehensive explanation of psychological functioning than examining each construct independently (Compas et al., 2001; Nicholls et al., 2009).

Furthermore, existing studies have predominantly focused on team-sport athletes or mixed sport samples, whereas evidence from endurance sports remains scarce (Nicholls and Polman, 2007). Endurance running presents unique psychological demands characterized by prolonged individual effort, self-regulation, and sustained exposure to performance-related stressors. These sport-specific characteristics may influence the relationships between self-esteem, anxiety, and coping strategies differently than in other athletic populations (Nicholls and Polman, 2007). In addition, adolescence represents a critical developmental period during which psychological resources and coping mechanisms are still evolving, making the examination of these associations particularly relevant (Compas et al., 2001). Recent evidence has also highlighted adolescence as a period of increased vulnerability to mental health challenges in athletes, emphasizing the importance of identifying psychological factors that may protect against stress-related difficulties and support positive adaptation (Rice et al., 2016; Gustafsson et al., 2017).

Another important gap concerns the role of gender in the interplay between self-esteem, anxiety, and coping. While some studies have reported gender-related differences in anxiety levels and preferred coping strategies (Craft et al., 2003; Weinberg and Gould, 2019), findings remain inconsistent (Tamminen and Gaudreau, 2014). Consequently, little is known about whether gender influences the relationships among these psychological constructs in adolescent athletes. Examining these associations may contribute to a more nuanced understanding of psychological adaptation in competitive sport and help identify gender-specific needs for psychological support.

Finally, evidence from North African countries remains limited, restricting the generalizability of current knowledge across diverse cultural settings. Cultural norms, social expectations, and sport participation experiences may shape athletes’ self-perceptions, emotional responses, and coping behaviors (Tamminen and Gaudreau, 2014; Weinberg and Gould, 2019). Consequently, investigating these relationships among Tunisian junior runners not only expands the geographical scope of sport psychology research but also provides an opportunity to determine whether patterns observed in Western populations are replicated within a distinct cultural and sporting context.

Therefore, the present study aimed to examine the associations between self-esteem, anxiety, and coping strategies among Tunisian junior runners, while also exploring potential gender differences in these relationships. We hypothesized that self-esteem would be negatively associated with anxiety and positively associated with adaptive coping strategies, whereas anxiety would be positively related to maladaptive coping strategies and negatively related to adaptive coping (Craft et al., 2003; Nicholls et al., 2009). We further expected gender-related differences in these associations, with female athletes reporting higher anxiety and greater reliance on emotion-focused or avoidance coping, and male athletes demonstrating greater use of problem-focused coping (Craft et al., 2003; Weinberg and Gould, 2019). By simultaneously examining self-esteem, anxiety, and coping strategies within a population of adolescent endurance athletes a group that remains underrepresented in the literature this study seeks to provide novel insights into the mechanisms underlying psychological adaptation to competitive stress and to inform targeted interventions aimed at promoting athletes’ well-being, mental health, and performance (Rice et al., 2016; Gustafsson et al., 2017).

## Methods

### Study design

The present study will adopt a cross-sectional descriptive and correlational design to examine the relationships between self-esteem, anxiety, and coping strategies among Tunisian junior runners, with a focus on potential gender differences. Data will be collected at a single point in time from a sample of junior athletes using standardized self-report questionnaires measuring self-esteem, competitive anxiety, and coping strategies. This design is appropriate for identifying associations between psychological variables and comparing groups (male vs. female athletes) without manipulating any independent variables. Statistical analyses will include descriptive statistics, Pearson correlation analysis to assess relationships among variables, and inferential tests (e.g., *t*-tests or MANOVA) to examine gender differences, as well as regression analyses to explore predictive relationships between self-esteem, anxiety, and coping strategies.

### Participants

This study involved a total of 156 Tunisian junior athletics runners specializing in middle- and long-distance events, who voluntarily participated in the study. The sample included 76 male athletes (48.7%) and 80 female athletes (51.3%), ensuring a relatively balanced representation of gender. Participants were aged between 18 and 19 years, with a mean age of 18.38 years (SD = 0.488), indicating a homogeneous age group typical of junior competitive athletes. In terms of training load, athletes reported an average weekly training duration of 6.04 h (SD = 1.452), reflecting regular engagement in structured endurance training programs.

Participants were recruited using a non-probabilistic quota sampling technique, which allowed the researchers to ensure proportional representation of male and female athletes within the target population. This approach was chosen due to the practical constraints of accessing competitive athletes and the need to obtain a balanced sample across gender categories. Inclusion criteria required participants to be actively engaged in middle- or long-distance running at a junior competitive level and to have consistent training participation. All participants were informed about the objectives of the study and participated voluntarily, ensuring ethical compliance and confidentiality of responses.

Eligibility criteria required athletes to be actively engaged in competitive sport and regularly participating in official competitions. Participants were recruited using a non-probability quota sampling method. Prior to participation, all athletes were informed about the aims of the study, the voluntary nature of participation, and the confidentiality of their responses. Written informed consent was obtained from all participants and from parents or legal guardians for minors in accordance with ethical guidelines for research involving human participants. This study was conducted following the latest version of the Declaration of Helsinki and the protocol was approved by the local ethics committee of the National Centre of Medicine and Science of Sports, Tunis (CNMSS-LR09SEP01) before the commencement of the study. None of the participating athletes had a history of psychological and/or musculoskeletal, neurological, or orthopedic disorders 6 months prior to the start of the study.

### Procedure

Initially, official authorization was obtained from the Technical Director of the Tunisian Athletics Federation to conduct the study with competitive athletes. Following approval, the selected athletics clubs were contacted and informed about the purpose, objectives, and procedures of the research. Consent was subsequently obtained from both the clubs and the participating athletes.

An agreement was established with the clubs to administer the research instruments in an online format. Prior to participation, athletes were informed that their involvement was voluntary and anonymous and that all collected data would be treated confidentially in accordance with Tunisian data protection regulations.

All participants received standardized instructions to ensure consistent completion of the questionnaires. Data collection was conducted on a single occasion during the main competitive phase of the 2021–2022 athletics season, specifically during regional and national competitions in which the athletes were officially participating. The questionnaires were completed approximately 1–2 h before the start of competition, a period considered sufficiently close to the competitive event to capture athletes’ pre-competition psychological states while minimizing interference with their preparation routines.

The study employed a cross-sectional design; therefore, assessments were conducted at a single time point rather than repeatedly across the season. This approach was chosen to provide a standardized evaluation of the relationships among self-esteem, competitive anxiety, and coping strategies under comparable competitive conditions for all participants. Nevertheless, it is acknowledged that anxiety and coping responses may vary according to the timing and importance of competitions. Consequently, future longitudinal studies should examine these psychological variables at multiple stages of the season and across competitions of varying competitive significance.

### Measures

#### Demographic information

A demographic questionnaire was used to collect basic participant information, including age, sex, years of athletic experience, and weekly training volume (hours of practice per week). These variables were included to describe the sample and control for potential confounding factors related to training exposure and sport experience.

#### Competitive state anxiety inventory-2 revised (CSAI-2R)

Pre-competitive anxiety was assessed using the French version of the Competitive State Anxiety Inventory-2 Revised (CSAI-2R), validated by Martinent et al. (2010). The original instrument, the CSAI-2, developed by Martens et al. (1990), is one of the most widely used measures of situational anxiety in sport settings. It was designed to capture the multidimensional nature of competitive anxiety, including cognitive anxiety, somatic anxiety, and self-confidence.

However, several psychometric limitations of the original CSAI-2 have been reported in the literature, particularly regarding construct validity and the ambiguity of certain items (e.g., the term “concern” in the cognitive anxiety subscale) (Burton and Naylor, 1997; Lane et al., 1999). In response to these concerns, Cox et al. (2003) developed the CSAI-2 Revised (CSAI-2R), which has demonstrated improved factorial validity and reliability across multiple validation studies (Cox et al., 2003; Lundqvist and Hassmén, 2005; Martinent et al., 2010).

The French version used in this study consists of 16 items measuring three dimensions: cognitive anxiety, somatic anxiety, and self-confidence. Participants rate each item on a Likert-type scale, with higher scores indicating greater levels of anxiety or self-confidence depending on the subscale. The CSAI-2R has demonstrated satisfactory internal consistency in French populations, with Cronbach’s alpha coefficients ranging from 0.76 to 0.83 across subscales (Martinent et al., 2010).

#### Inventory of coping strategies in sports competition (ISCCS)

Coping strategies were measured using the Inventory of Coping Strategies in Sports Competition (ISCCS) developed by Gaudreau and Blondin (2002). This instrument contains 39 items distributed across 10 subscales: mental imagery, thought control, effort expenditure, seeking support, relaxation, logical analysis, venting of unpleasant emotions, disengagement, social withdrawal, and mental distraction.

These subscales are further grouped into three higher-order dimensions: task-oriented coping, disengagement coping, and distraction coping. Responses are rated on a 5-point Likert scale ranging from 1 (never) to 5 (always). The ISCCS is widely recognized as a robust instrument for assessing coping in competitive sport contexts and is considered one of the most appropriate tools for quantitative analysis of coping strategies in sport psychology research (Crocker et al., 2015).

The scale has demonstrated strong psychometric properties across multiple studies, confirming its validity and reliability in different athletic populations (Gaudreau and Blondin, 2002; Amiot et al., 2004; Gaudreau et al., 2010). Further theoretical support for its use is provided by research linking coping processes with coping self-efficacy and affective responses in sport settings (Ntoumanis and Biddle, 1998; Nicholls et al., 2011).

#### Rosenberg self-esteem scale (RSES)

Self-esteem was assessed using the Rosenberg Self-Esteem Scale (RSES), one of the most widely used instruments for measuring global self-worth (Rosenberg, 1965). The scale consists of 10 items evaluating both positive and negative feelings about the self. Participants respond using a 4-point Likert scale ranging from 1 (strongly disagree) to 4 (strongly agree), with higher total scores indicating higher self-esteem. Total scores range from 10 to 40.

In the present study, the validated French version of the RSES was used (Vallières and Vallerand, 1990). The scale has demonstrated strong psychometric qualities across diverse populations and cultural contexts (Gray-Little et al., 1997; Sinclair et al., 2010). Reported reliability indices are consistently satisfactory, with Cronbach’s alpha values typically ranging between 0.75 and 0.85 across studies (Rosenberg, 1965; Martín-Albo et al., 2007).

### Statistical analyses

Statistical analyses were performed using IBM SPSS Statistics version 25 (IBM Corp., 2017). Data were first screened for missing values, accuracy, and normality. Normality was evaluated using skewness and kurtosis indices, in line with recommendations for moderate sample sizes, with values within ±2 considered acceptable (Field, 2013; Tabachnick and Fidell, 2013).

Descriptive statistics (means, standard deviations, minimum–maximum values), reliability coefficients (Cronbach’s *α*) (Nunnally and Bernstein, 1994), and distribution indices were computed for all study variables (Table 1). Independent-samples t-tests were used to examine gender differences in coping strategies, competitive anxiety, and self-esteem (Table 2). Effect sizes were reported using Cohen’s *d* (Cohen, 1988).

**Table 1:** Descriptive statistics, reliability coefficients, and normality indices for the ISCCS, CSAI-2R, and RSES.

Variable	Scale range	Min	Max	Skewness	Kurtosis	Mean ± SD	Cronbach’s *α*
ISCCS							
Thought control	1–20	4	19	−0.08	−1.34	11.35 ± 3.99	0.725
Mental imagery	1–20	4	20	−0.00	−1.45	12.00 ± 4.75	0.875
Relaxation	1–20	4	12	0.40	−0.25	7.92 ± 2.04	0.712
Effort expenditure	1–15	2	15	0.33	−1.27	7.92 ± 4.03	0.702
Logical analysis	1–20	4	20	−0.05	−1.52	11.95 ± 4.79	0.877
Seeking support	1–20	4	14	0.28	−0.58	8.53 ± 2.34	0.703
Venting of unpleasant emotions	1–20	4	18	0.50	−0.96	9.26 ± 3.88	0.859
Disengagement	1–20	4	19	0.44	−1.15	9.18 ± 4.15	0.800
Mental distraction	1–20	4	20	0.27	−1.22	10.40 ± 4.47	0.873
Social withdrawal	1–20	4	15	1.14	0.79	7.38 ± 2.75	0.705
Task-oriented coping	30–115	30	82	−0.28	−0.97	59.66 ± 12.52	0.915
Disengagement coping	8–40	8	37	0.41	−1.15	18.44 ± 7.78	0.912
Distraction coping	8–40	8	34	0.45	−0.97	17.38 ± 6.81	0.849
CSAI-2R							
Cognitive anxiety	5–20	6	19	0.21	−1.33	12.36 ± 3.57	0.911
Somatic anxiety	6–24	7	21	0.27	−1.26	12.97 ± 3.74	0.882
Self-confidence	5–20	7	20	−0.02	−1.56	13.10 ± 3.76	0.904
RSES							
Self-esteem	10–40	16	40	−0.16	−0.34	29.54 ± 4.51	0.876

All skewness values ranged from −0.28 to 1.14 and all kurtosis values ranged from −1.56 to 0.79, indicating acceptable normality. Cronbach’s α coefficients ranged from 0.702 to 0.915, demonstrating acceptable-to-excellent internal consistency.

**Table 2:** Gender differences in coping strategies, anxiety dimensions, and self-esteem.

Variable	Female (*n* = 80) mean ± SD	Male (*n* = 76) mean ± SD	*t*	*p*	Cohen’s *d*
Mental imagery	11.26 ± 4.58	12.78 ± 4.84	−2.01	0.046	0.32
Effort expenditure	8.90 ± 4.14	6.88 ± 3.66	3.22	0.002	0.52
Logical analysis	11.18 ± 4.71	12.76 ± 4.77	−2.09	0.038	0.33
Distraction coping	18.71 ± 6.90	15.99 ± 6.48	2.54	0.012	0.41
Cognitive anxiety	13.24 ± 3.50	11.43 ± 3.43	3.25	0.001	0.52
Self-confidence	12.30 ± 3.63	13.93 ± 3.74	−2.77	0.006	0.44
Self-esteem	28.25 ± 3.93	30.91 ± 4.71	−3.84	0.001	0.62

Cohen’s *d* values of 0.20, 0.50, and 0.80 indicate small, moderate, and large effect sizes, respectively.

Pearson product–moment correlations were used to examine associations among self-esteem, competitive state anxiety dimensions, and coping strategies (Table 3).

**Table 3:** Pearson correlations among higher-order coping dimensions, anxiety dimensions, self-confidence, and self-esteem.

Variable	1	2	3	4	5	6	7
Cognitive anxiety	–	0.827**	−0.850**	−0.781**	0.806**	0.686**	−0.495**
Somatic anxiety		–	−0.793**	−0.728**	0.769**	0.636**	−0.439**
Self-confidence			–	0.804**	−0.782**	−0.699**	0.500**
Task-oriented coping				–	−0.863**	−0.645**	0.463**
Disengagement coping					–	0.787**	−0.491**
Distraction coping						–	−0.518**
Self-esteem							–

Strong associations were observed between anxiety dimensions and coping profiles (|*r*| = 0.64–0.81), ***indicating that anxiety and confidence are closely linked to athletes’ coping responses during competition. The symbol (**) indicates a statistically significant difference from the pre-intervention value at *p* < 0.05.

Given the high intercorrelation between cognitive and somatic anxiety (*r* = 0.83), indicating severe multicollinearity (VIF > 38), multivariate analysis of variance (MANOVA) was used to examine the combined effects of cognitive anxiety, somatic anxiety, and self-confidence on coping dimensions (task-oriented, disengagement, and distraction coping) (UCLA Institute for Digital Research and Education, 2020). A separate MANOVA was conducted for self-esteem (Table 4). Wilks’ Lambda (*Λ*) was used as the primary multivariate test statistic, with partial eta-squared (*η*^2^_p_) reported as the effect size.

**Table 4:** Multivariate analysis of variance (MANOVA) for coping dimensions.

Effect	Wilks’ *Λ*	*F*	df₁	df₂	*p*	*η*^2^ₚ
Cognitive anxiety	0.90	5.46	3	150	0.001	0.10
Somatic anxiety	0.94	3.00	3	150	0.032	0.06
Self-confidence	0.81	11.85	3	150	<0.001	0.19
Self-esteem	0.71	20.29	3	152	<0.001	0.29

*η*^2^ₚ = partial eta-squared. The multivariate effect of anxiety dimensions and self-confidence on the combined coping dimensions was significant, Wilks’ *Λ* = 0.18, *F*(9, 448.6) = 38.99, *p* < 0.001, *η*^2^ₚ = 0.44. The multivariate effect of self-esteem was significant, Wilks’ *Λ* = 0.71, *F*(3, 152) = 20.29, *p* < 0.001, *η*^2^ₚ = 0.29.

Multiple linear regression analyses were conducted to examine the predictive effects of cognitive anxiety, somatic anxiety, self-confidence, and self-esteem on coping strategies (Tables 5, 6). Multicollinearity was assessed using tolerance and variance inflation factor (VIF), with VIF > 10 and tolerance < 0.10 indicating severe multicollinearity (Hair et al., 2019). As confirmed in Table 7, strong multicollinearity between anxiety dimensions limited the interpretability of individual regression coefficients.

**Table 5:** Hierarchical multiple regression analyses predicting coping dimensions from anxiety dimensions and self-confidence.

Dependent variable	Predictor	*B*	SE	95% CI [*LL*, *UL*]	*β*	*t*	*p*	VIF	Tolerance	Δ*R*^2^
Task-oriented coping	(Intercept)	59.66	6.12	[47.57, 71.75]	–	9.75	<0.001	–	–	
	Cognitive anxiety	−1.03	0.33	[−1.68, −0.39]	−0.28	−3.15	0.002	**38.41**	**0.026**	
	Somatic anxiety	−0.37	0.27	[−0.90, 0.16]	−0.11	−1.37	0.174	**39.59**	**0.025**	
	Self-confidence	1.55	0.29	[0.99, 2.12]	0.47	5.42	<0.001	3.96	0.252	**0.67**
Disengagement coping	(Intercept)	18.44	3.90	[10.73, 26.15]	–	4.73	<0.001	–	–	
	Cognitive anxiety	0.81	0.21	[0.40, 1.23]	0.37	3.89	<0.001	**38.41**	**0.026**	
	Somatic anxiety	0.51	0.17	[0.17, 0.85]	0.25	2.97	0.003	**39.59**	**0.025**	
	Self-confidence	−0.56	0.18	[−0.92, −0.20]	−0.27	−3.06	0.003	3.96	0.252	**0.70**
Distraction coping	(Intercept)	17.38	4.12	[9.24, 25.52]	–	4.22	<0.001	–	–	
	Cognitive anxiety	0.49	0.22	[0.06, 0.93]	0.26	2.23	0.027	**38.41**	**0.026**	
	Somatic anxiety	0.24	0.18	[−0.12, 0.60]	0.14	1.34	0.182	**39.59**	**0.025**	
	Self-confidence	−0.68	0.19	[−1.06, −0.30]	−0.38	−3.51	<0.001	3.96	0.252	**0.54**

*N* = 156. CI, confidence interval; LL, lower limit; UL, upper limit. VIF, variance inflation factor. Bold VIF values indicate severe multicollinearity (VIF > 10; tolerance < 0.10). The anxiety dimensions (cognitive and somatic) share 83% variance (*r* = 0.83), creating multicollinearity that inflates standard errors and renders individual regression coefficients unstable. Adjusted *R*^2^ values: task-oriented coping = 0.70, disengagement coping = 0.70, distraction coping = 0.54. Overall model statistics: task-oriented coping, *F*(3, 152) = 122.48, *p* < 0.001; disengagement coping, *F*(3, 152) = 119.05, *p* < 0.001; distraction coping, *F*(3, 152) = 61.27, *p* < 0.001.

**Table 6:** Simple regression analyses predicting coping dimensions from self-esteem.

Dependent variable	Predictor	*B*	SE	95% CI [*LL*, *UL*]	β	*t*	*p*	Adjusted *R*^2^
Task-oriented coping	Self-esteem	1.31	0.19	[0.93, 1.69]	0.48	6.77	<0.001	0.22
Disengagement coping	Self-esteem	−0.85	0.12	[−1.09, −0.61]	−0.49	−7.00	<0.001	0.24
Distraction coping	Self-esteem	−0.75	0.10	[−0.95, −0.55]	−0.51	−7.31	<0.001	0.25

*N* = 156. CI, confidence interval; LL, lower limit; UL, upper limit. All models significant at *p* < 0.001. Self-esteem was not multicollinear with anxiety dimensions (all *r*s < 0.50) and produced stable, interpretable coefficients.

**Table 7:** Regression assumption checks.

Assumption	Test	Statistic	*p*	Decision
Independence of errors	Durbin-Watson	1.41–1.43	—	Acceptable (1 < DW < 3)
Normality of residuals	Jarque-Bera	10.14–10.74	0.005–0.006	Some departure; robust to moderate violation with *N* = 156
Homoscedasticity	Breusch-Pagan	9.74–9.81	0.020–0.021	Moderate heteroscedasticity; bootstrap CIs reported
Multicollinearity	VIF (cognitive anxiety)	38.41	—	**Severe violation (VIF > 10)**
Multicollinearity	VIF (somatic anxiety)	39.59	—	**Severe violation (VIF > 10)**
Multicollinearity	VIF (self-confidence)	3.96	—	Acceptable (VIF < 5)

VIF, variance inflation factor. Bootstrap 95% CIs (10,000 resamples) were computed to address heteroscedasticity concerns. Bold values represent statistically significant effects (*p* < 0.05).

Regression assumptions were assessed using standard diagnostic procedures, including inspection of residual plots for linearity and homoscedasticity, histograms and normal probability plots for normality of residuals, and the Durbin–Watson statistic for independence of errors. Where heteroscedasticity was indicated, bias-corrected and accelerated (BCa) bootstrap confidence intervals (10,000 resamples) were computed.

Standardized (*β*) and unstandardized (B) coefficients, standard errors, confidence intervals, *t*-values, *p*-values, and adjusted *R*^2^ values were reported. In addition, relative importance of predictors was assessed using semipartial *R*^2^ (Δ*R*^2^) (Johnson and LeBreton, 2004). Given the presence of multicollinearity among anxiety variables, interpretation focused primarily on model-level statistics (*R*^2^, *F*-tests), MANOVA results, and relative importance estimates rather than individual beta weights.

Statistical significance was set at *p* < 0.05 for all analyses.

## Results

### Preliminary analyses

Descriptive statistics, reliability coefficients, and normality indices are presented in Table 1. All scales demonstrated acceptable to excellent internal consistency (Cronbach’s *α* = 0.70–0.92), and skewness and kurtosis values indicated acceptable normality. Gender differences in key variables are reported in Table 2.

As shown in Tables 3, 5, cognitive and somatic anxiety were highly correlated (*r* = 0.83), resulting in severe multicollinearity (VIF = 38.41–39.59; tolerance ≈ 0.025). In contrast, self-confidence showed acceptable collinearity (VIF = 3.96). This issue was further confirmed in regression diagnostics (Table 7), which indicated instability of anxiety-related coefficients. Accordingly, interpretation of regression findings focused on overall model fit, MANOVA results, and relative importance indices.

### Correlation analyses

Pearson correlations are presented in Table 3. Cognitive and somatic anxiety were strongly positively associated with maladaptive coping strategies, including disengagement (*r* = 0.79 and 0.73, respectively), distraction (*r* = 0.69 and 0.64), and venting of emotions (*r* = 0.77 and 0.76), and negatively associated with adaptive coping strategies such as logical analysis (*r* = −0.85 and −0.79), mental imagery (*r* = −0.80 and −0.77), and thought control (*r* = −0.76 and −0.73) (all *p* < 0.01).

In contrast, self-confidence showed the opposite pattern, being positively associated with adaptive coping (logical analysis *r* = 0.85; mental imagery *r* = 0.83; task-oriented coping *r* = 0.80) and negatively associated with maladaptive coping (disengagement *r* = −0.78; distraction *r* = −0.70) (all *p* < 0.01). Self-esteem showed weaker but consistent associations, being positively related to task-oriented coping (*r* = 0.46) and negatively related to disengagement (*r* = −0.49) and distraction coping (*r* = −0.52) (Table 3).

### Multivariate analysis of variance

The MANOVA examining the effects of cognitive anxiety, somatic anxiety, and self-confidence on coping dimensions was significant, Wilks’ *Λ* = 0.18, *F*(9, 448.6) = 38.99, *p* < 0.001, *η*^2^_p_ = 0.44 (Table 4). Significant multivariate effects were found for cognitive anxiety (*η*^2^_p_ = 0.10), somatic anxiety (*η*^2^_p_ = 0.06), and self-confidence (*η*^2^_p_ = 0.19). Self-esteem also showed a significant multivariate effect, Wilks’ *Λ* = 0.71, *F*(3, 152) = 20.29, *p* < 0.001, *η*^2^_p_ = 0.29.

### Regression analyses

Regression results are summarized in Tables 5, 6. The models including cognitive anxiety, somatic anxiety, and self-confidence explained substantial variance in task-oriented coping (*R*^2^ = 0.71), disengagement coping (*R*^2^ = 0.70), and distraction coping (*R*^2^ = 0.55) (Table 5). However, severe multicollinearity between anxiety dimensions (VIF > 38) indicated unstable regression coefficients (Table 7), and therefore interpretation emphasized model-level indices and relative importance.

Self-confidence emerged as the strongest predictor of task-oriented coping (*β* = 0.47, *p* < 0.001) and distraction coping (*β* = −0.38, *p* < 0.001), accounting for 71.3 and 64.6% of explained variance, respectively. Cognitive anxiety was the main predictor of disengagement coping (*β* = 0.37, *p* < 0.001; 45.4% of explained variance), while somatic anxiety showed weaker and less consistent effects.

For task-oriented coping, cognitive anxiety was negatively associated (*β* = −0.28, *p* = 0.002), whereas somatic anxiety was non-significant. For disengagement coping, all predictors were significant, whereas for distraction coping only self-confidence remained a stable predictor.

As shown in Table 6, self-esteem significantly predicted all coping dimensions. Higher self-esteem was associated with greater task-oriented coping (*β* = 0.48, *R*^2^ = 0.22) and lower disengagement (*β* = −0.49, *R*^2^ = 0.24) and distraction coping (*β* = −0.51, *R*^2^ = 0.25), all *p* < 0.001.

## Discussion

The present study investigated the relationships among self-esteem, competitive state anxiety, and coping strategies in Tunisian junior runners, while also examining potential gender differences. The findings revealed that anxiety, self-confidence, and self-esteem were strongly associated with the way athletes coped with competitive stress. More importantly, the results suggest that coping behavior is not merely a response to situational demands but is closely linked to athletes’ psychological resources and perceptions of competence. This interpretation is consistent with the transactional model of stress and coping, which posits that individuals’ cognitive appraisals of stressful situations influence both emotional responses and coping behaviors (Lazarus and Folkman, 1984; Nicholls et al., 2016).

One of the most notable findings was the strong negative association between cognitive anxiety and task-oriented coping strategies, particularly logical analysis and mental imagery. Cognitive anxiety reflects worries, doubts, and concerns about performance outcomes (Martens et al., 1990). Athletes experiencing elevated levels of cognitive anxiety may therefore devote attentional resources to threat-related thoughts rather than to effective coping processes. This interpretation is supported by attentional control theory, which suggests that anxiety impairs cognitive efficiency by disrupting attentional regulation and increasing susceptibility to distracting thoughts (Eysenck et al., 2007). In practical terms, junior runners who are preoccupied with concerns about performance or failure may be less able to engage in adaptive coping strategies such as planning, imagery, and rational problem solving.

The strong positive associations observed between anxiety and disengagement-related coping strategies are also noteworthy. Rather than simply indicating that anxious athletes cope less effectively, these findings suggest a potentially self-reinforcing cycle in which anxiety promotes avoidance-oriented coping, which subsequently reduces perceptions of control and further intensifies stress responses. Previous research has shown that avoidance and disengagement coping strategies are generally associated with poorer emotional adjustment and less effective stress management in sport (Gaudreau and Blondin, 2004; Compas et al., 2001). This mechanism may be particularly relevant in endurance running, where athletes must sustain effort and concentration over extended periods while coping with fatigue and performance uncertainty.

Conversely, self-confidence emerged as one of the strongest predictors of adaptive coping. Athletes with greater self-confidence reported higher levels of logical analysis, mental imagery, and task-oriented coping. These findings are consistent with previous studies showing that self-confidence facilitates positive performance appraisals and promotes the use of problem-focused coping strategies (Nicholls et al., 2009; Weinberg and Gould, 2019). From a theoretical perspective, self-confidence may function as a psychological resource that increases perceived control over competitive situations, thereby encouraging athletes to engage actively with stressors rather than avoid them (Bandura, 1997).

An important contribution of the present study is the distinction between self-confidence and self-esteem. Although both variables were associated with adaptive coping profiles, anxiety-related variables explained a larger proportion of variance in coping strategies than self-esteem alone. This finding suggests that self-esteem may represent a relatively stable personal resource, whereas competitive anxiety and self-confidence are more proximal psychological states that exert a stronger influence on coping behavior immediately before competition. Similar distinctions between trait-like and state-like psychological factors have been proposed in previous sport psychology research (Rosenberg, 1965; Crocker et al., 2015). Consequently, interventions aimed at improving coping effectiveness may benefit from targeting both global self-worth and competition-specific confidence.

The observed gender differences also warrant careful consideration. Female athletes reported higher cognitive anxiety and lower self-confidence and self-esteem than male athletes. Although these findings are generally consistent with previous meta-analytic evidence indicating higher levels of competitive anxiety among female athletes (Craft et al., 2003), they should not be interpreted as reflecting inherent gender characteristics. Psychological responses to competition are shaped by multiple factors, including socialization processes, coaching interactions, perceived competence, and cultural expectations (Tamminen and Gaudreau, 2014). In the Tunisian context, sociocultural influences may affect how male and female athletes experience and express competitive stress. Therefore, future research should further explore the contextual factors underlying these gender differences.

The regression analyses provide additional insight into the mechanisms underlying coping behavior. Anxiety-related variables explained between 51 and 69% of the variance in coping dimensions, highlighting the central role of competitive anxiety in shaping athletes’ responses to stress. Notably, cognitive anxiety was a stronger predictor of coping than somatic anxiety, suggesting that athletes’ interpretations and evaluations of competitive situations may be more influential than physiological symptoms alone. This finding supports multidimensional anxiety theory, which emphasizes the importance of cognitive processes in determining performance-related outcomes (Martens et al., 1990; Cox et al., 2003).

Given that data were collected approximately 1–2 h before official regional and national competitions, the observed relationships likely reflect psychological processes occurring in a meaningful competitive context. However, anxiety levels are known to fluctuate according to competition importance, performance expectations, and seasonal timing (Jones et al., 1994; Hanton et al., 2008). Consequently, the present findings should be interpreted as representing a snapshot of athletes’ psychological functioning during a specific phase of competition rather than stable characteristics across an entire season. Longitudinal studies assessing athletes at multiple competitive time points are therefore needed to determine the stability of these relationships.

From an applied perspective, the findings suggest that interventions designed to reduce cognitive anxiety and strengthen self-confidence may enhance athletes’ ability to use adaptive coping strategies under pressure. Psychological skills training programs incorporating imagery, relaxation, self-talk, and attentional control techniques have demonstrated effectiveness in improving athletes’ stress management and performance (Vealey, 2007; Weinberg and Gould, 2019). Coaches and sport psychologists should therefore prioritize the development of psychological resources that promote perceptions of competence, control, and resilience in young athletes.

Several limitations should be acknowledged. First, the cross-sectional design prevents causal inference regarding relationships among self-esteem, anxiety, and coping strategies. Second, reliance on self-report measures may introduce social desirability and response bias. Third, data were collected at a single time point during the competitive season, limiting the ability to capture temporal fluctuations in psychological states across different competitive contexts. Fourth, severe multicollinearity was observed between cognitive and somatic anxiety (*r* = 0.83; VIF > 38), leading to unstable regression estimates and limiting interpretation of their unique effects (Field, 2018). Accordingly, interpretation focused on overall model fit, relative importance indices, and MANOVA results (Table 4), which confirmed that anxiety dimensions jointly predict coping profiles, although their specific contributions cannot be statistically disentangled. Future studies should consider composite anxiety scores or latent variable modeling to address this issue. Fifth, confirmatory factor analysis (CFA) was not conducted. Although the ISCCS, CSAI-2R, and RSES have established validity in previous research (e.g., Gaudreau and Blondin, 2002; Martinent et al., 2010; Rosenberg, 1965; Vallières and Vallerand, 1990), future work should include measurement model testing, particularly in Tunisian athletic populations and cross-cultural adaptations.

Finally, the sample was restricted to Tunisian junior runners, limiting generalizability to other sports, performance levels, and cultural contexts. Future research should adopt longitudinal and multi-method designs to examine the dynamic interplay between self-esteem, anxiety, and coping across training and competition. Additional work should explore psychosocial factors such as resilience, emotional regulation, motivational climate, and coaching behaviors to better explain inter-individual differences in stress adaptation. Such approaches would advance understanding of psychological functioning in youth sport and inform evidence-based interventions to enhance both performance and well-being.

## Conclusion

The present study investigated the relationships between self-esteem, competitive state anxiety, and coping strategies among Tunisian junior runners, with a particular focus on gender differences. The findings demonstrated that these psychological constructs are strongly interrelated. Higher levels of self-esteem and self-confidence were associated with more adaptive, task-oriented coping strategies, whereas higher levels of cognitive and somatic anxiety were linked to greater use of maladaptive coping strategies such as disengagement, distraction, and emotional venting. In addition, significant gender differences were observed, with male athletes generally reporting higher self-esteem and more adaptive coping patterns, while female athletes exhibited higher anxiety levels and a greater tendency toward emotion-focused and avoidance coping strategies. Overall, the results highlight the central role of psychological factors in how young athletes manage competitive stress.

From a practical perspective, these findings emphasize the importance of integrating psychological skills training into youth athletics programs, particularly for middle- and long-distance runners who are regularly exposed to sustained performance pressure. Coaches and sport psychologists should focus on developing athletes’ self-esteem and self-confidence through positive feedback, mastery-oriented goal setting, and reinforcement of personal improvement rather than solely outcome-based success. In addition, training programs should include coping skills interventions such as relaxation techniques, mental imagery, thought control strategies, and structured problem-solving approaches to help athletes manage pre-competition anxiety more effectively. Special attention may also be needed for female athletes, who appear to be more vulnerable to anxiety and less adaptive coping patterns in this sample, suggesting the value of gender-sensitive psychological support strategies.

Future research should adopt longitudinal designs to better understand how self-esteem, anxiety, and coping strategies evolve over time and influence athletic performance across different stages of development. Experimental studies could also be useful in evaluating the effectiveness of psychological interventions aimed at enhancing coping skills and reducing competitive anxiety. Furthermore, expanding research to include athletes from different sports, competitive levels, and cultural contexts would improve the generalizability of findings. Finally, incorporating additional psychological constructs such as resilience, mental toughness, motivation, and emotional regulation could provide a more comprehensive understanding of the psychological mechanisms underlying stress management in youth sport.

## Data Availability

The raw data supporting the conclusions of this article will be made available by the authors, without undue reservation.
